# Impact of blood hypercoagulability on in vitro fertilization outcomes: a prospective longitudinal observational study

**DOI:** 10.1186/s12959-017-0131-7

**Published:** 2017-03-28

**Authors:** Grigoris T. Gerotziafas, Patrick Van Dreden, Emmanuelle Mathieu d’Argent, Eleftheria Lefkou, Matthieu Grusse, Marjorie Comtet, Rabiatou Sangare, Hela Ketatni, Annette K. Larsen, Ismail Elalamy

**Affiliations:** 1 Cancer Biology and Therapeutics, Centre de Recherche Saint-Antoine, Institut National de la Santé et de la Recherche Médicale (INSERM) U938 and Université Pierre et Marie Curie (UPMC), Sorbonne Universities, Paris, France; 2 Service d’Hématologie Biologique, Hôpital Tenon, Hôpitaux Universitaires Est Parisien, Assistance Publique Hôpitaux de Paris, 4, rue de la Chine, Paris, Cedex 20 France; 3 Clinical Research Department, Diagnostica Stago, Gennevilliers, France; 4 Department of Obstetrics and Gynecology, Hôpital Tenon, Hôpitaux Universitaires Est Parisien, Assistance Publique Hôpitaux de Paris, Paris, France

**Keywords:** Tissue factor, Blood coagulation tests, Thrombin generation, In vitro fertilization, Hypercoagulability

## Abstract

**Background:**

Blood coagulation plays a crucial role in the blastocyst implantation process and its alteration may be related to in vitro fertilization (IVF) failure. We conducted a prospective observational longitudinal study in women eligible for IVF to explore the association between alterations of coagulation with the IVF outcome and to identify the biomarkers of hypercoagulability which are related with this outcome.

**Methods:**

Thirty-eight women eligible for IVF (IVF-group) and 30 healthy, age-matched women (control group) were included. In the IVF-group, blood was collected at baseline, 5–8 days after administration of gonadotropin-releasing hormone agonist (GnRH), before and two weeks after administration of human follicular stimulating hormone (FSH). Pregnancy was monitored by measurement of *β*HCG performed 15 days after embryo transfer. Thrombin generation (TG), minimal tissue factor-triggered whole blood thromboelastometry (ROTEM®), procoagulant phospholipid clotting time (Procoag-PPL®), thrombomodulin (TMa), tissue factor activity (TFa), factor VIII (FVIII), factor von Willebrand (FvW), D-Dimers and fibrinogen were assessed at each time point.

**Results:**

Positive IVF occurred in 15 women (40%). At baseline, the IVF-group showed significantly increased TG, TFa and TMa and significantly shorter Procoag-PPL versus the control group. After initiation of hormone treatment TG was significantly higher in the IVF-positive as compared to the IVF-negative group. At all studied points, the Procoag-PPL was significantly shorter and the levels of TFa were significantly higher in the IVF-negative group compared to the IVF-positive one. The D-Dimers were higher in the IVF negative as compared to IVF positive group. Multivariate analysis retained the Procoag-PPL and TG as predictors for the IVF outcome.

**Conclusions:**

Diagnosis of women with hypercoagulability and their stratification to risk of IVF failure using a model based on the Procoag-PPL and TG is a feasible strategy for the optimization of IVF efficiency that needs to be validated in prospective trials.

## Background

The link between blood hypercoagulability and infertility or in vitro fertilization (IVF) failure is a puzzling issue. Hypercoagulability could be intrinsic or caused by the hormone treatment preceding the IVF procedure [1–6]. Tissue factor is the major trigger of blood coagulation and thrombin generation is the ultimate step that leads to fibrin formation. The activation of coagulation induced by tissue factor (TF) expressed by perivascular decidualized human endometrial stromal cells is an essential part of the mechanism that favors blastocyst implantation and prevents peri-implantational hemorrhage during endovascular trophoblast invasion. Thrombin generation is required for cell proliferation, neoangiogenesis, trophoblast invasion and remodeling of the spiral arteries and arterioles [7–9]. Thus, the shift of blood coagulation equilibrium towards locally enhancement of thrombin generation may have some beneficial effects for a positive outcome of IVF. On the other hand, in infertile women activation or dysfunction of platelets, endothelial cells and monocytes has been observed [10, 11].

Newer laboratory assays allow the assessment of global blood coagulation and clot formation process. Among them, the Calibrated Automated Thrombogram® and the minimal TF-triggered whole blood thromboelastometry allow the evaluation of thrombin generation and clot formation processes [12–14]. The measurement of the procoagulant phospholipid dependent clotting time (Procoag-PPL®) reflects the plasma concentration of procoagulant membrane vesicles of cellular origin [15, 16]. Biomarkers of endothelial cell activation such as thrombomodulin activity (TMa) and TF activity (TFa) measured in plasma offer information on the status of the endothelial cells at the vasculature.

The aim of the present prospective, observational longitudinal study was to identify biomarkers of hypercoagulability which could have some predictive value for the IVF outcome. Thrombin generation, clot formation kinetics and molecular biomarkers of cellular hypercoagulability were assessed at women eligible for IVF at baseline (before any hormone treatment administration), at the down-regulation phase of the menstrual cycle and after ovarian stimulation. Biochemical diagnosis of pregnancy was the end-point of the study.

## Methods

### Study design and participants

A monocentric prospective, non-interventional cohort study was designed. From June 2014 to June 2015 blood samples were obtained from 38 women eligible for IVF (IVF-group). Women were recruited at the baseline consultation and then they were followed until pregnancy test was performed. Biochemical diagnosis of pregnancy was the end-point of the study. According to the levels of β-chorionic gonadotropin (βHCG) women were stratified into two subgroups: IVF-positive if βHCG levels were higher than 100 IU/L and IVF-negative if (βHCG) levels were equal or lower than 100 IU/L. The evolution of the pregnancy was not recorded.

The control group consisted of 30 healthy, age-matched women, without any known hereditary or acquired thrombophilic alteration or personal history of thrombotic or bleeding disorder who had undergone at least one uneventful physically conceived pregnancy and without any personal history of miscarriage. The protocol of the study was in accordance with the commitment of the Helsinki declaration and was approved by the institutional ethics committee. All subjects provided informed written consent before inclusion in the study.

#### Inclusion criteria

Women were eligible for IVF according to established selection criteria applied in our institution. All women had full blood count, platelet count, prothrombin time, activated partial thromboplastin time, fibrinogen, renal and liver function within the normal range.

#### Exclusion criteria

Women younger than 18 years or older than 45 years, weight less than 50 kg or more than 100 kg, with a personal or family history of venous thromboembolism (VTE) or hemorrhagic syndromes, known hereditary or acquired thrombophilia, active anticoagulant or antiplatelet treatment or use of these agents during the last 30 days before inclusion, hospitalization for any reason within the previous 3 months, abnormal full blood count or platelet count and ongoing cardiovascular, renal or liver disease, malignancy, or arterial hypertension, known systematic or chronic disease (autoimmune syndrome, heart disease, severe or uncontrolled thyroid disease or HIV infection), treatment with non-steroid anti-inflammatory drugs within the last 10 days before inclusion, ovarian insufficiency (FSH > 9 IU/ml and/or number of antral follicles <8) or polycystic ovary syndrome (defined according to the Rotterdam criteria).

### Hormone treatment for the artificial reproductive technique

Estrogen production was first down-regulated to induce controlled ovarian stimulation. Three different protocols of down regulation were used: a long gonadotropin-releasing hormone (GnRH) agonist or a short agonist or an antagonist. Ovarian stimulation was done with recombinant human follicular stimulating hormone (FSH) at doses ranging from 75 IU to 450 IU per day depending on age, body mass index (BMI), antral follicle count, size and number of follicles and estradiol levels (E2). This stimulation was initiated once pituitary desensitization had been achieved (E2 level <50 pg/mL). The response was followed by E2 measurement six days later and by ultrasound scanning of the ovarian follicles at days 9–10 after the first FSH injection, and repeated when necessary. Transvaginal oocyte retrieval was scheduled 35 to 36 h after human recombinant chorion gonadotrophin (hCG) injection and embryo transfer was performed 2–3 days later. On day 2, individually cultured embryos were evaluated on the basis of the number of blastomeres, blastomere size, fragmentation rate and presence of multinucleated blastomeres. Therefore, the ovocytes were retrieved 10 to 14 days after starting the stimulation with the FSH.

### Outcomes

Achievement of biochemical pregnancy was the outcome of the study. Pregnancy was controlled by quantitative measurement of *β*HCG 15 days after embryo transfer.

### Blood sampling

Blood samples were collected before the administration of any hormone treatment (T0) and during the IVF procedure as follows: at the maximal down-regulation of the menstrual cycle; between the 5^th^ and 8^th^ day from the administration of the GnRH agonist (T1); at maximal stimulation after treatment by FSH and before hCG injection (T2) and two weeks after gonadotropin-releasing hormone (GnRH) injection (T3). Blood samples were obtained by atraumatic puncture of the antecubital vein, using a 20-gauge needle without tourniquet, into siliconized vacutainer tubes containing 0.105 mol/L trisodium citrate; 1/9 v/v (Becton and Dickinson, France). Platelet-poor plasma (PPP) was obtained by double centrifugation at 2000 *g* for 20 min at room temperature and plasma aliquots were stored at −80 °C until assayed. Samples were assessed within two weeks after collection. Thromboelastometry was carried out with fresh whole blood.

### Molecular and functional analysis

***Thrombin generation assay*** **.** Thrombin generation in plasma was assessed using the Calibrated Automated Thrombogram assay (CAT®, Diagnostica Stago, Asnières France) according to manufacturers’ instructions, in the presence of optimal concentrations of TF (5 pM) and procoagulant phospholipids (4 μM) using the PPP-Reagent®. Assay’s performance has been published elsewhere [12, 17].

***Minimal TF-triggered whole blood thromboelastometry (min TF-WB TEM)*** was assessed in citrated whole blood, on the ROTEM® instrument (TEM®, Munich, Germany). Thromboelastometry was performed at 37 °C in citrated fresh whole blood within 30 min after veinipuncture using 5 pM of TF as described elsewhere [18]. The following parameters of the thromboelastometric trace were analyzed: (a) *Clotting time* (CT, in sec): time from the start of the sample run to the point of first significant clot appearance corresponding to an amplitude of 2 mm, (b) *Clot formation time* (CFT, in sec): time from CT until the level of clot firmness reaches an arbitrary value of 20 mm, (c) *α-angle* (degree): measurement of clot development kinetics, (d) *Maximum clot firmness* (MCF in mm): the maximum vertical amplitude of the thromboelastogram.

***Procoagulant phospholipid-dependent clotting time (Proag-PPL)*** was measured with STA^®^Procoag-PPL, (Diagnostica Stago, Asnières, France) according to the manufacturer’s instructions as described elsewhere. The inter- and intra-assay coefficients of variation were 3 and 4% respectively.

***Thrombomodulin activity***. Plasma levels of thrombomodulin activity (TMa) were measured with a functional test on the STA-R analyzer (Diagnostica Stago, Asnières, France) as described elsewhere [19]. The inter- and intra-assay coefficients of variation were 5 and 6% respectively.

***Specific TF activity***. Tissue Factor activity (TFa) in PPP was measured with a clotting-based assay as previously described [20, 21]. The inter- and intra-assay coefficients of variation were 7% and 5% respectively. The levels of, FVIII, FvW, D-Dimers and fibrinogen were measured with conventional assays according to the manufacturer’s instructions (Diagnostica Stago, Asnières, France).

### Statistical analysis

The calculation of the sample size was based on the minimum number of patients required for a significant power for the detection of differences (a) between the IVF and control group, (b) at the IVF at the studied time points, (c) between IVF positive and IVF negative groups. The minimum sample size of 27 individuals for each group (IVF and control as well as IVF at each time point) was defined to warrant a two-tail significance at the limit of 5% and a prediction power of 95% with a two-sided α level of 0.05. Regarding the sub-group analysis (IVF positive and IVF negative) the minimum size of 15 patients for each group warrants two-tail significance at the limit of 5% and a prediction power of 85% with a two-sided α level of 0.05.

Special effort and attention was given to avoid missing values. The data are presented as mean ± sd. The Mann–Whitney test for independent samples was used for the comparisons of the studied parameters between the IVF and the control group and between the IVF-positive and IVF-negative group. Non-parametric Wilcoxon test for related samples and ANOVA test were applied to compare changes in variables at the studied time points during the observation period. Pearsons’ test was applied to control correlation between thrombogram parameters and studied blood coagulation variables. Dichotomous variables were compared with *χ* ^2^ test. The Upper Normal Limit (UNL) and the Lower Normal Limit (LNL) for each parameter of the studied variables were defined in the control group as follows: UNL = mean + 2 SD, and LNL: = mean – 2 SD. The UNL and LNL of the studied biomarkers were defined in the control group and were compared to the corresponding normal reference range used by our laboratory. The normal ranges have been established according to the requirements for the good quality of laboratory practice by performing the tests in healthy individuals representative of the general population regarding age, sex, ethnicity, BMI. Two-sided values of *p* < 0.05 were considered as statistically significant.

The model development started by defining the positive diagnosis of pregnancy (if βHCG levels were equal or lower than 100 IU/L) as the dependent variable. The first step consisted of the univariate analysis in order to identify the variables associated with positive pregnancy. The selection of independent variables (which are the biomarkers of hypercoagulability) was done at the level of 5% using the stepwise procedure. The multivariable linear regression model was used to explore the effect of the independent variables on pregnancy outcome. The variables, found to be significant in the univariate analysis (*p* < 0.05) were included in the multivariate analysis. The variable with the highest p value was excluded from the model. The discrimination capacity of the model was tested with receiver operating characteristics (ROC) analysis and the area under the curve (AUC) was calculated for the quantification of the discrimination capacity of the model. The SPSS statistical software package (Chicago inc 6.1) was used for statistical analysis. Values are mean ± standard deviation.

## Results

In total 38 women were eligible for the study and completed the follow up. The clinical and demographic characteristics of the studied groups are shown in Table 1. Positive IVF occurred in 15 women (40%) and negative in 23 (60%). The demographic characteristics and the indication for IVF were similar in the IVF-positive and IVF-negative group. None of the women included in the IVF and the control group had hypertension, hyperlipidemia or diabetes. None of the women suffered thromboembolic complication.

**Table 1:** Demographic and clinical characteristics of women eligible for IVF and in subgroups stratified according to the IVF outcome

Characteristics	IVF group (*n* = 38)	IVF-positive (*n* = 15)	IVF-negative (*n* = 23)	Control group (*n* = 30)
Demographics				
Age (years)	33.7 ± 3.5	33.7 ± 3.5	33.7 ± 3.5	32.2 ± 2.2
BMI	22.8 ± 2.9	22.8 ± 2.9	22.8 ± 2.9	23.8 ± 2.1
Indication for IVF				
endometriosis	2	1	1	–
idiopathic	17	17	14	–
ovarian insufficiency	1	1		–
tubal	4	2	2	–
male origin	14	7	7	–
Previous pregnancy				
0	31	15	16	–
1	4	3	1	10
2	3	1	2	20
Previous miscarriages				
0	36	17	19	30
1	2	1	1	0
Cardiovascular risk factors				
Current smokers	7 (18%)	3 (10%)	4 (13%)	8 (27%)

The UNL and LNL of the studied biomarkers in the control group were not significantly different as compared to the respective normal reference ranges used in our laboratory (Table 2).

**Table 2:** Baseline profile of hypercoagulability in women eligible for IVF and dynamic changes of the global coagulation tests and molecular biomarkers of hypercoagulability during hormone treatment in the cohort of women eligible for IVF and according to the IVF outcome

	Normal reference range	Control (*n* = 30)	IVF T0			IVF T1			IVF T2			IVF T3		
			All women (*n* = 38)	IVF-positive (*n* = 15)	IVF-negative (*n* = 23)	All women (*n* = 38)	IVF-positive (*n* = 15)	IVF-negative (*n* = 23)	All women (*n* = 38)	IVF-positive (*n* = 15)	IVF-negative (*n* = 23)	All women (*n* = 38)	IVF-positive (*n* = 15)	IVF-negative (*n* = 23)
Lag time (min)	2.1–3.8	3.41 ± 0.24 UNL: 3.89 LNL: 2.93	2.88 ± 0.59***	2.94 ± 0.59	2.84 ± 0.59	2.64 ± 0.58°°°	2.60 ± 0.62	2.65 ± 0.58	2.64 ± 0.67^өөө^	2.68 ± 0.86	2.62 ± 0.56	2.51 ± 0.56^+++^	2.87 ± 0.66	2.39 ± 0.53
ttPeak (min)	4.0–6.6	6.48 ± 0.32 UNL: 7.12 LNL: 5.84	5.58 ± 1.06***	5.68 ± 1.07	5.51 ± 1.07	5.23 ± 0.9°°°	4.97 ± 0.80	5.35 ± 0.93	5.03 ± 0.99 ^өөө$^	4.95 ± 1.24	5.08 ± 0.85	4.79 ± 1.03^+++^	5.54 ± 1.6	4.54 ± 0.81
ETP (nM.min)	1178–1600	1408 ± 80 UNL: 1568 LNL: 1248	1939.65 ± 646***	1992.10 ± 736.60	1903.89 ± 592.17	1744.52 ± 549.19°°°	2006.95 ± 569.74	1619.55 ± 505.49^§^	1950.54 ± 551.03 ^өөө$^	2172.95 ± 539.59	1819.71 ± 529.65^*^	1536.38 ± 371.53^+^	1931 ± 388.91	1471.5 ± 315.68^§^
Peak (nM)	222–330	262 ± 25 UNL: 312 LNL: 212	352.3 ± 100***	359.55 ± 109.08	347.35 ± 95.34	330.32 ± 99.07°°°	399.89 ± 96.32	297.19 ± 83.51^§^	372.79 ± 82.11 ^өөө$L^	415.89 ± 73.08	347.44 ± 78.16 ^*^	317.69 ± 80.33^+++^	355.69 ± 56	305.02 ± 58.28^§^
MRI (nM/min)	60–120	86.59 ± 14.10 UNL: 115 LNL: 58	135.27 ± 44***	135.76 ± 47.86	134.93 ± 42.66	132.62 ± 50.3°°°	173.17 ± 50.21	113.31 ± 38.03^§^	159.77 ± 44.53 ^өөө$L^	188.83 ± 48.09	142.67 ± 32.90 ^*^	146.31 ± 49.47^+++^	152.66 ± 98	144.2 ± 29.95^§^
CT (sec)	140–307	245.57 ± 45.61 UNL: 335 LNL: 155	269.53 ± 74.47	251.53 ± 78.8	281.26 ± 70.79	233.78 ± 65.71	206.07 ± 41.44	251.41 ± 72.77^§^	236.40 ± 62.41	209.07 ± 42.35	254.62 ± 67.70^§^	204.43 ± 23.65^+++^	216.8 ± 7.66	197.56 ± 27.03^§^
CFT (sec)	6–155	80.46 ± 37.26 UNL: 155 LNL: 6	92.82 ± 28.43	93.2 ± 37.25	92.57 ± 21.82	89.94 ± 26.60	88.43 ± 26.73	90.91 ± 27.1	88.14 ± 21.68	86.79 ± 16.79	89.05 ± 24.78	79.79 ± 23.51	64.8 ± 13.08	88.11 ± 24.38^§^
α angle (°)	55–92	72.86 ± 7.35 UNL: 58 LNL: 88	71.76 ± 5.02	71.60 ± 6.25	71.87 ± 4.18	72.78 ± 4.29	72.86 ± 4.13	72.73 ± 4.48	72.89 ± 3.83	73.57 ± 3.03	72.43 ± 4.28	74.14 ± 4.35	76.8 ± 2.59	72.67 ± 4.53
MCF (mm)	54–74	65.86 ± 4.31 UNL: 75 LNL: 57	64.63 ± 7.59	60.67 ± 10.22	63.91 ± 5.1	64.47 ± 4.48	64.50 ± 4.69	64.45 ± 4.45	64.31 ± 5.02	64.03 ± 5.5	64.29 ± 4.82	66.64 ± 5.39	69 ± 3.81	65.33 ± 5.87
Procoag-PPL (sec)	42–85	60.21 ± 9.3 UNL: 79 LNL: 42	33.0 ± 5.36***	36.45 ± 5.83	30.53 ± 3.34^§^	35.5 ± 7.04°°°	41.37 ± 7.55	32.27 ± 4.18^§^	34.14 ± 4.71 ^өөө^	37.45 ± 4.51	32.12 ± 3.63^*^	39.4 ± 6.45^+++^	41.09 ± 5.79	38.32 ± 6.88
TFa (pM)	0.02–0.25	0.24 ± 0.11 UNL: 0,46 LNL: 0,02	0.47 ± 0.31***	0.32 ± 0.23	0.59 ± 0.31^§^	0.40 ± 0.32°°	0.21 ± 0.19	0.51 ± 0.32^§^	0.42 ± 0.27 ^өөө^	0.22 ± 0.15	0.55 ± 0.26^*^	0.4 ± 0.14^+++^	0.41 ± 0.18	0.45 ± 0.12
TMa (%)	50–126	90 ± 18 UNL: 126 LNL: 54	127.56 ± 61.54***	121.60 ± 38.38	131.81 ± 74.50	131 ± 50.54°°°	113.17 ± 54.49	133.86 ± 44.96	119.90 ± 39.26 ^өөө^	103.55 ± 34.07	129.89 ± 39.72^§^	126.9 ± 34.27^+++^	122.43 ± 7.74	129.82 ± 44.01
FVIII (%)	50–150	105 ± 19 UNL: 143 LNL: 67	101.31 ± 29.22	98.73 ± 27.50	103.14 ± 30.92	117 ± 40.45	115.25 ± 34.90	113.73 ± 28.86	140.10 ± 28.30 ^өөөL^	152.73 ± 24.71	132.39 ± 28.17^*^	117.7 ± 22.97^+LL^	133.57 ± 14.08	107.55 ± 22.10^§^
vWF (%)	50–150	105 ± 21 UNL: 147 LNL: 63	99.44 ± 20.08	95.53 ± 14.31	102.24 ± 23.30	125.17 ± 23.11°°°^£^	116.08 ± 18.73	108.23 ± 21.50	129.14 ± 21.37 ^өөө^	137.09 ± 24.77	124.28 ± 18.03	113.4 ± 16.79		
F g (g/l)	1.8–4.0	2.9 ± 0.4 UNL: 3.7 LNL: 2.1	3.18 ± 0.8	3.01 ± 0.7	3.28 ± 0.86	2.98 ± 0.64	3.02 ± 0.9	2.96 ± 0.48	3.02 ± 0.65	3.10 ± 0.9	2.98 ± 0.51	2.9 ± 0.57	3.17 ± 0.64	2.71 ± 0.47
D-Dimers (μg/ml)	<0.50	0.25 ± 0.12 UNL: 0,49 LNL: 0.01	0.29 ± 0.12	0.29 ± 0.11	0.31 ± 0.13	0.30 ± 0.09	0.30 ± 0.09	0.3 ± 0.11	0.56 ± 0.28 ^өөөL^	0.39 ± 0.11	0.68 ± 0.31 ^*^	0.7 ± 0.4^+++LL^	0.49 ± 0.15	0.85 ± 0.45^*^

*UNL* upper normal limits, *LNL* lower normal limits, *ETP* the endogenous thrombin potential, *Peak* the peak concentration of thrombin, *ttPeak* time to reach the peak concentration of thrombin, *MRI* mean rate index of thrombin generation, *CT* clotting time, *CFT* clot formation time, *MCF* maximum clot firmness, *AUC* area under curve, *α angle* reflecting the fibrin polymerization rate, *Procoag-PPL* procoagulant phospholipid dependent clotting time, *FVIII* factor VIII, *D-Di* D-Dimer, *TMa* thrombomodulin activity, *vWF* von Willebrand factor, *TFa* tissue factor activity, *Fg* fibrinogen

*p < 0.05; **p < 0.01; ***p < 0.001, T0 versus control

°*p* < 0.05; °° *p* < 0.01; °°°*p* < 0.001, T1 versus control

^ө^ *p* < 0.05; ^өө^ *p* < 0.01; ^өөө^ *p* < 0.001, T2 versus control

^+^ *p* < 0.05 ; ^++^ *p* < 0.01 ; ^+++^ *p* < 0.001, T3 versus control

^£^ *p* < %0.05 T1 versus T0

^L^ *p* < 0.05 T2 versus T1

^LL^ *p* < 0.05 T3 versus T2

^§^ *p* < 0.05; IVF-negative versus IVF-positive

### Baseline profile

At T0, the IVF-group showed significantly increased thrombin generation (marked by shorter lag-time and ttPeak and higher ETP, Peak and MRI), and significantly shorter Procoag-PPL as compared to the control group. At least one thrombogram parameter was higher than the UNL in 50% of women and lower than the LNL in 14%. The IVF group also had significantly higher levels of TFa, TMa. The levels of FVIII, FvW, D-Dimers and fibrinogen were not significantly different between the IVF-group and the control group (Table 2).

### Effect of down-regulation of the menstrual cycle

At T1, thrombin generation, Procoag-PPL*,* TFa, TMa, FVIII, fibrinogen and D-Dimers were not significantly different as compared to T0. The levels of FvW significantly increased as compared to T0 (Table 2). Women with ETP, Peak or MRI above the UNL at T0 did not show any significant differences at T1.

### Effect of ovarian stimulation

At T2, Peak, MRI and the levels of FVIII and D-Dimers were significantly increased as compared to T1 (Table 2). In contrast the Procoag-PPL and the levels of TFa, TMa and FvW did not vary significantly as compared to T1.

### Effect of GnRH treatment

At T3, thrombin generation was not significantly different as compared to T2. The Procoag-PPL, TFa and TMa, FvW, and fibrinogen remained at the same levels as in T2. The levels of FVIII significantly decreased at T3 as compare to T2. The levels of D-Dimers significantly increased as compared to T2 (Table 2).

At all studied time points the parameters of thromboelastometry were not significantly different between the IVF-group and the control group (Table 2).

### Blood hypercoagulability and IVF outcome

At T2 and T3 the ETP, Peak and MRI were significantly higher in the IVF-positive as compared to the IVF-negative group. At T0, T1 and T2 the Procoag-PPL was significantly shorter in the IVF-negative group compared to the IVF-positive group. At all time points except T3, the levels of TFa were significantly higher in the IVF-negative group as compared to the IVF-positive group. The levels of TMa were significantly higher in the IVF-negative group as compared to the IVF-positive group only at T2. At T2 and T3 FVIII levels were significantly lower in IVF negative as compared to IVF positive. At T2 and T3, D-Dimers were significantly higher in the IVF-negative as compared to the IVF-positive group. At T1 and T2, the CT was significantly shorter in the IVF-positive group as compared to the IVF-negative. (Table 2).

The Procoag-PPL was inversely correlated with the lag-time (*r* = −0.304; *p* = 0.013), ETP (*r* = −0,4; *p* = 0.001) and MRI (*r* = −0.380; *p* = 0.002) as well as with the TFa (*r* = −0.399; *p* = 0.001). Thrombomodulin was inversely correlated with ETP (*r* = −0.346; *p* = 0.019).

Univariate analysis showed the following biomarkers to have a significant correlation with the positive outcome of IVF: at T0, the FvW; at T1 the ETP, Peak, MRI, Procoag-PPL and TFa; at T2, the ETP, Peak, MRI, Procoag-PPL, TFa and D-Dimers (Table 3).

**Table 3:** Univariate analysis and odds ratio of thrombin generation test and the biomarkers of hypercoagulability for the positive IVF outcome

Variables	T0		T1		T2		T3	
	odds ratio (95% Conf. Interval)	*p*	odds ratio (95% Conf. Interval)	*p*	odds ratio [95% Conf. Interval)	*p*	odds ratio (95% Conf. Interval)	*p*
lag-time (min)	1.288 (0.39–4.17)	0.672	0.855 (1.96–3.71)	0.835	1.583 (0.54–4.64)	0.403	2.564 (0.11–54.54)	0.56
ETP (nM.min)	0.999 (0.99–1.00)	0.936	1.002 (1.00–1.00)	0.047*	1.002 (1.00–1.003)	0.016*	1.000 (0.99–1.00)	0.625
Peak (nM)	0.999 (0.6–2.2)	0.974	1.013 (1.00–1.02)	0.006*	1.018 (1.01–1.03)	0.004*	1.001 (0.98–1.02)	0.952
ttPeak (min)	1.148 (0.59–2.23)	0.684	0.529 (0.13–2.16)	0.375	1.034 (0.40–2.66)	0.945	1.944 (0.42–8.93)	0.394
MRI (nM/min)	0.997 (0.98–1.01)	0.792	1.032 (1.01–1.05)	0.003*	1.037 (1.01–1.06)	0.005*	0.993 (0.96–1.02)	0.715
Procoag-PPL (sec)	1.009 (0.88–1.15)	0.895	1.399 (1.07–1.82)	0.013*	1.421 (1.08–1.86)	0.011*	1.112 (0.91–1.33)	0.32
TFa (pM)	0.329 (0.04–2.96)	0.322	0.0022 (0–0.21)	0.009*	4.60e–08 (2.14e–13.00)	0.007*	1.141 (1.03–1.27)	0.009
TMa (%)	0.997 (0.99–1.00)	0.598	0.987 (0.96–1.01)	0.223	0.977 (0.95–1.00)	0.107	0.993 (0.96–1.01)	0.368
FVIII (%)	1.003 (0.97–1.02)		1.004 (0.97–1.03)	0.799	1.028 (0.99–1.06)	0.137	1.143 (0.97–1.34)	0.119
VWF (%)	1.052 (1.01–1.08)	0.004*	1.009 (0.98–1.04)	0.578	1.012 (0.07–1.05)	0.581	1.062 (0.97–1.16)	0.166
Fibrinogen (g/L)	1.228 (0.48–3.16)	0.67	0.979 (0.18–5.16)	0.981	2.014 (0.31–13)	0.465	6.346 (0.63–63.38)	0.116
D-Dimers (μg/ml)	0.997 (0.99–1.00)	0.408	1 (0.99–1.00)	0.951	0.992 (0.98–0.99)	0.016*	1.243 (0.82–1.45)	0.234

There was a strong association between IVF-positive outcome and Procoag-PPL longer than 31.1 sec. Women who had Procoag-PPL between 31.1 and 54.9 sec had 24-fold higher probability for pregnancy than the women with Procoag-PPL values lower than 31.1 sec.

Multivariate analysis for the identification of biomarkers which are correlated with a positive outcome of IVF retained the Procoag-PPL and MRI of thrombin generation. Multivariate analysis applied at the T1 led to the construction of a prediction model including the Procoag-PPL and the MRI according to the equation:

$$ Y=\left( 0.043* MRI\right)*\left( 0.45* Procoag\mathit{\hbox{-}} PPL\right) $$

**Fig. 1:**
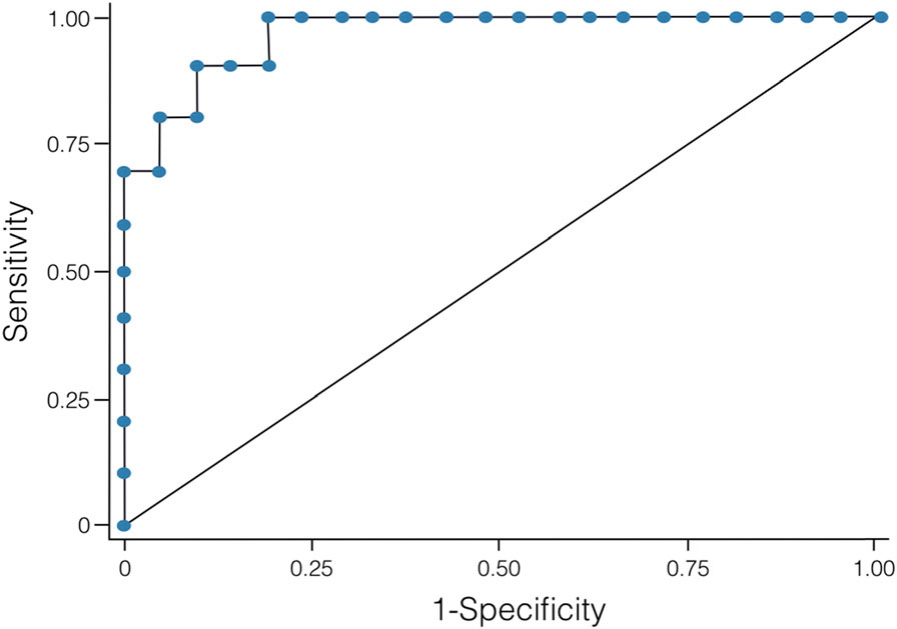
The ROC analysis of the prediction model of the IVF outcome based on the MRI and Procoag-PPL. The AUC is 0.9667

The ROC curve showed a very good specificity and sensitivity of the model for the prediction of pregnancy since the area under the curve was 0.99 (Fig. 1).

## Discussion

The present prospective longitudinal observational study demonstrates that at baseline, women eligible for IVF present blood hypercoagulability which is characterized by significant increase of platelet and endothelial cell activation biomarkers. The baseline state of cellular hypercoagulability, which persisted practically unchanged during the period of hormone treatment administration for IVF, was consisted of significantly shortening of Procoag-PPL clotting time. This test is correlated with increased concentration of procoagulant microparticles derived from platelets or other cells [22, 23]. The levels of TFa and TMa, at baseline and during hormone treatment, were also significantly increased as compared to age-matched women with naturally occurring uneventful pregnancies. The TFa and TMa are biomarkers of endothelial cell or platelet activation [24–26]. In addition, increased TFa levels in plasma is a marker of monocyte activation [27]. The Procoag-PPL, TFa and TMa levels were not significantly modified during treatment for estrogen down-regulation and ovarian stimulation indicating that the shift towards cellular hypercoagulability observed at baseline was not correlated with hormone variations. The data from the present study are in accordance with those recently published by Olausson et al. which demonstrate that platelet, endothelial and monocyte-derived microparticles and inflammation biomarkers are significantly increased in women undergoing IVF [28]. Thrombin generation was also significantly enhanced but the baseline levels of FVIII, FvW, D-Dimers and fibrinogen were similar to those observed in the control group, confirming previous studies [4]. To the best of our knowledge, this is the first study showing that infertility is linked to a systemic cell activation that offers procoagulant substances, mainly endothelial cells and platelets. This concept is supported by recent studies reporting that increased levels of plasminogen activator inhibitor 1 (PAI-1), thrombin activatable fibrinolysis inhibitor (TAFI) or tissue factor pathway inhibitor (TFPI), which are synthesized and secreted by activated endothelial cells are related with infertility and IVF failure [29–32].

Furthermore, the present study showed that short Procoag-PPL clotting time as well as increased TFa and TMa levels are independent risk factors for IVF failure. The implication of high levels of TF and procoagulant microparticles in the pathogenesis of infertility, IVF failure and vascular complications during pregnancy, such as recurrent first trimester miscarriage, fetal loss, stillbirth, early and severe pre-eclampsia or prematurity has already been reported by others (reviewed in [33, 34]). Herein, we demonstrate for a first time that the shortened Procoag-PPL associated with the mean rate index (MRI) of the propagation phase of thrombin generation assessed at the maximal down-regulation of the menstrual cycle (between the 5^th^ and 8^th^ day from the administration of the GnRH agonist) are predictors of IVF outcome. These tests could be used in the construction of a risk assessment model for IVF issue. The design of the present study does not allow the identification of the underlying causes that lead to cellular activation and hypercoagulability which is observed at baseline. This investigation appears to be attractive for the elucidation of the link with hypercoagulability in women eligible for IVF. The association between hypercoagulablity and negative IVF outcome has recently been reported by Di Nisio et al., who proposed that the increase of D-Dimers levels in plasma is a predictor for IVF failure [35]. The implication of increased D-Dimers in the sterility is further supported by the data presented by Di Micco et al. [36]. The univariate analysis of the data reported herein confirms that the increase of D-Dimers during hormone treatment is a negative prognostic factor for IVF outcome. However, the impact of D-Dimers disappeared in the multivariate analysis indicating that high concentrations of procoagulant phospholipids, detected by the short Procoag-PPL, have a dominant role in negative IVF outcome.

Interestingly, increased thrombin generation was found to be a positive predictor for IVF outcome. This finding is in accordance with the concept that thrombin generation is necessary for blastocyst implantation, remodeling of decidualized human endometrial stromal cells and subsequent trophoblast invasion and remodeling of the spiral arteries and arterioles; a process driven by TF expression in the endometrial microenvironment which is in contact with mother’s blood [7–10]. The concept that the non-suffering of endothelial cells, platelets or other cells that potentially release procoagulant phospholipids, is determinant for a positive IVF outcome is supported by our study. Indeed, the presence of high levels of procoagulant phospholipids in plasma, as expressed by shortened Procoag-PPL was the dominant parameter related with IVF failure while the decrease of thrombin generation was found to be a complementary factor. Control measurements showed that factor V and factor II levels were within the normal range (data not shown) ruling out clotting factors’ consumption. The reasons for which thrombin generation is decreased in women with IVF failure have to be investigated.

At all studied time points the parameters of thromboelastometry were not significantly different between the IVF-group and the control group. Fibrinogen levels, platelet count and hematocrit are variables with a major impact on thromboelastometric profile. Both IVF-group and the control group had these variables within the normal ranges.

We found that when the ovarian stimulation was maximal, thrombin generation, FVIII, FvW and D-Dimers levels were significantly increased, in agreement with older studies [5, 37–41]. However, these changes were not reflected on the kinetics of clot formation and its qualitative characteristics when coagulation was triggered by a low TF concentration. In women undergoing hormone treatment for IVF preparation thromboelastographic analysis performed after triggering contact system showed a slight but significant acceleration of the kinetics of clot formation following GnRH treatment indicating that peak concentrations of estrogens are associated with a possible enhancement of FXII activation [42]. Women who had baseline thrombin generation above the UNL remained at the same levels during the down-regulation and the stimulation phase of the treatment (data not shown). Therefore, our data, in agreement with previous studies [43] and support the concept that hormone treatment for IVF represents a mild procoagulant stimulus, which has a minor effect on the global haemostatic balance, the kinetics of clot formation or its qualitative characteristics. Two weeks after hCG injection and embryo transfer, thrombin generation, TMa, TFa and Procoag-PPL remained without any significant modifications as compared to the phase of ovarian hyperstimulation. In contrast, the D-Dimers tended to increase and this finding is in agreement with others [2]. De Nisio et al., found that one week after the administration of gonadotropin, D-Dimers levels increased considerably [35]. We also found that even two weeks after GnRH injection D-Dimers levels are still increased, reflecting enhanced fibrin formation or fibrinolysis following r-hCG, as described by Biron et al. [2].

The present study has some limitations. The number of the enrolled women, although it provides sufficient statistical power to identify the most relevant biomarkers related with IVF outcome, does not allow generalizability of the model. The group of IVF women was heterogeneous regarding the IVF indication. In some women the indication of the IVF was of male origin. Although the elimination of these cases did not significantly influence the final results, this does not warrant that the presence of this subpopulation had no impact on the results. A new prospective study in a larger population is planned to corroborate these findings. Women enrolled in the study were treated with three different protocols for estrogen down-regulation. The subgroup analysis according to the therapeutic protocol did not demonstrate any significant difference on the studied biomarkers among the subgroups (data not shown). Several different hormone treatments and protocols are applied for IVF preparation by the different IVF centers. Whether this protocol variability influences the kinetics of the studied biomarkers has to be investigated. However, the major findings of our study, which are (a) the presence of cell derived hypercoagulable state at the baseline, before any treatment administration and (b) the predictive value of the Procoag-PPL clotting time associated with the MRI of thrombin generation on IVF outcome are not influenced by the subsequent hormone treatments for ovarian stimulation. Although this study fulfils the criteria for the statistical power in the selection of the most clinically relevant biomarkers, a new prospective independent validation of this model is required in a larger multicenter study.

It has been suggested that heparin may improve the intrauterine environment in sub-fertile women, by enhancing growth factors to improve attachment of the embryo to the lining of the womb [44]. Thus low molecular weight heparin (LMWH) is often offered to women eligible for IVF as an adjunct treatment in an attempt to improve the probability for a positive outcome. However, a recent meta-analysis showed that it is unclear whether peri-implantation heparin administration in assisted reproduction treatment cycles improves clinical pregnancy rates in sub-fertile women [45]. The elaboration of a risk assessment model with the clinically relevant biomarkers of hypercoagulability proposed by the present study is of clinical interest in order to identify women who could benefit from the peri-implantation administration of antithrombotic treatment.

## Conclusions

The data presented herein show that hypercoagulability of cellular origin is the dominant biological process related with IVF failure. Hypercoagulabilty of cellular origin appears at the baseline, before any hormone treatment administration and it is not modified by this treatment. The administration of hormone treatment during the IVF preparation does not lead to substantial alteration of global coagulation process. Among the large variety of the studied biomarkers, the evaluation of both Procoag-PPL and MRI of thrombin generation after down-regulation of the menstrual cycle appear to be the most clinically relevant biomarkers for the identification of the women at risk of IVF failure.

## Abbreviations

AUC: Area under the curve
BMI: Body mass index
CAT: Calibrated Automated Thrombogram
CFT: Clot formation time
CT: Clotting time
E2: Estradiol
ETP: Endogenous thrombin potential
FSH: Follicular stimulating hormone
FVIII: Factor VIII
FvW: Factor von Willebrand
GnRH: Gonadotropin-releasing hormone agonist
hCG: Human recombinant chorion gonadotropin
IVF: In vitro fertilization
LNL: Lower Normal Limit
MCF: Maximum clot firmness
min TF-WB TEM: Minimal TF-triggered whole blood thromboelastometry
MRI: Mean rate index
PA1-1: Plasminogen activator inhibitor 1
PPP: Platelet poor plasma
Procoag-PPL: Procoagulant phospholipid clotting time
ROC: Receiver operating characteristics
TAFI: Thrombin activatable fibrinolysis inhibitor
TFa: Tissue factor activity
TFPI: Tissue factor pathway inhibitor
TG: Thrombin generation
TMa: Thrombomodulin activity
ttPeak: Time to Peak
UNL: Upper Normal Limit
VTE: Venous thromboembolism
